# The primase subunits of DNA polymerase α, PRIM1 and PRIM2, are required for the replication of the geminivirus tomato yellow leaf curl virus in the host plant

**DOI:** 10.17912/micropub.biology.000735

**Published:** 2023-01-05

**Authors:** Hua Wei, Rosa Lozano-Durán

**Affiliations:** 1 Department of Plant Biochemistry, Centre for Plant Molecular Biology (ZMBP), Eberhard Karls University, D-72076 Tübingen, Germany.; 2 Shanghai Center for Plant Stress Biology, CAS Center for Excellence in Molecular Plant Sciences, Chinese Academy of Sciences, Shanghai 201602, China.

## Abstract

Geminiviruses are causal agents of devastating diseases in crops. Geminiviral genomes are single-stranded (ss) circular DNA molecules that replicate in the nucleus of the infected cell through double-stranded (ds) intermediates by co-opting the plant DNA replication machinery. However, the identity of the plant DNA polymerases enabling geminiviral replication has remained largely elusive. Recently, we showed that DNA polymerase α mediates the ss-to-ds conversion of tomato yellow leaf curl virus (TYLCV), and is therefore essential for the infection. Here, we provide data indicating that the primase subunits of DNA polymerase α, PRIM1 and PRIM2, are also required for TYLCV replication.

**Figure 1. PRIM1 and PRIM2 are required for replication of the geminivirus TYLCV.**
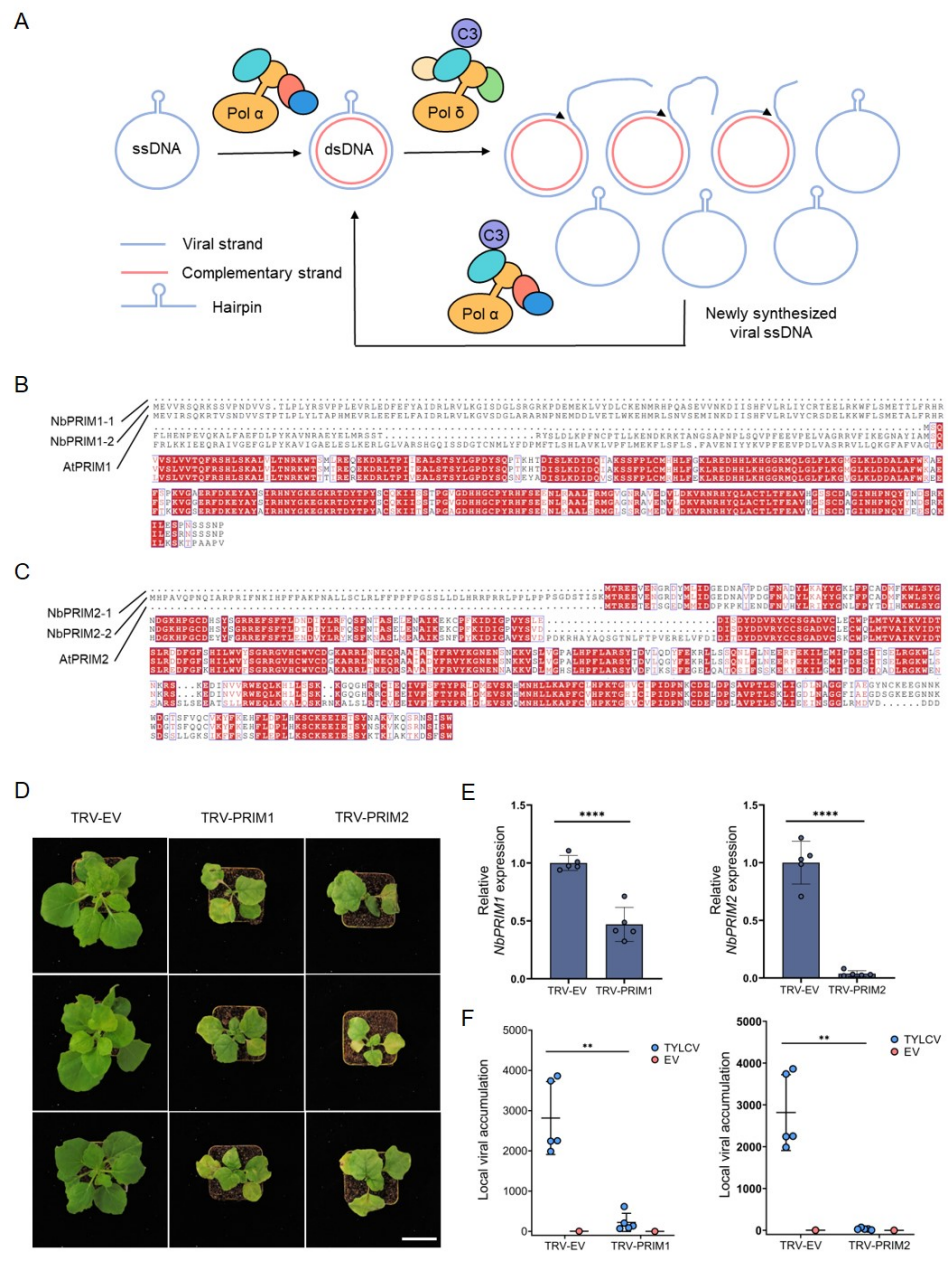
**(A)** Schematic representation of geminiviral replication. DNA polymerase α is required to convert the viral ssDNA genome to the dsDNA replicative intermediate, which is then replicated by DNA polymerase δ to produce new viral ssDNA. The virus-encoded C3 protein interacts with DNA polymerase α (POLA2 subunit) and DNA polymerase δ (POLD2 subunit), and enhances geminiviral replication probably through mediating selective recruitment of these holocomplexes to the viral genome. **(B** and **C)** Multiple sequence alignment of proteins encoded by *AtPRIM1* , *NbPRIM1* - *1* , and *NbPRIM1* - *2* or proteins encoded by *AtPRIM2* , *NbPRIM2* - *1* , and *NbPRIM2* - *2* (https://www.genome.jp/tools-bin/clustalw). **(D)** Developmental phenotypes of *NbPRIM1* - and *NbPRIM2* -silenced *N. benthamiana* plants. TRV empty vector (TRV-EV) is included as control. Images were taken at 2 weeks post-inoculation (wpi). Scale bar: 5 cm. **(E)** Silencing efficiency of *PRIM1* and *PRIM2* in TYLCV-inoculated *N. benthamiana* plants in (F). *NbPRIM1* - *1/2* and *NbPRIM2* -1/2 transcript accumulation in silenced and control plants, measured by RT-qPCR. *NbActin* was used as reference gene. Values are presented relative to those in the TRV-EV plants. Error bars represent SD with n=5 independent biological replicates. Samples were taken at 2 wpi. Asterisks indicate a statistically significant difference according to Student’s t test (****, P<0.0001). This experiment was repeated three times with similar results; results from one experiment are shown. **(F)** Viral accumulation in local TYLCV infections (3 days post-inoculation) in *NbPRIM1* -silenced, *NbPRIM2* -silenced, or control (TRV-EV) *N. benthamiana* plants measured by qPCR. Plants inoculated with the empty vector (EV) are used as negative control. Error bars represent SD with n=5 independent biological replicates. The 25S ribosomal DNA interspacer ( *ITS* ) was used as reference gene; values are represented relative to *ITS* . Asterisks indicate a statistically significant difference according to Student’s *t* -test (**, P<0.01). These experiments were repeated three times with similar results; results from one experiment are shown.

## Description

Geminiviruses are plant DNA viruses that cause devastating diseases in cash and staple crops worldwide. Geminiviral genomes are circular, single-stranded (ss) DNA molecules that replicate in the nucleus of the infected cell; these viruses, however, do not encode their own DNA polymerase, and hence fully rely on the co-option of the DNA replication machinery from the host cell. Despite the economic and humanitarian relevance of this viral family, the identity of the host proteins enabling the replication of their genome remains mostly elusive.

During the viral replication cycle, the viral ssDNA (viral strand, VS) has to be converted into a dsDNA intermediate (VS plus complementary strand, CS), which then serves as template for the subsequent rolling-circle replication (RCR); RCR results in the massive production of new ssDNA copies of the viral genome (Fig 1A). Only one viral protein, Rep (for replication-associated) is required for viral replication, with another virus-encoded protein, C3, acting as an enhancer in this process. Rep reprograms the cell cycle, recruits the DNA replication machinery to the viral genome, and nicks and ligates the viral DNA at the beginning and end of RCR (Wu et al., 2021). Recently, we identified two plant nuclear DNA polymerases from the B-family, DNA polymerase (DNA pol) α and DNA pol δ, as required for geminiviral replication: DNA pol α mediates the initial ss-to-ds conversion step, while DNA pol δ is expendable for this process but essential for RCR (Wu et al., 2021). At the onset of RCR, C3 might act selectively recruiting DNA pol δ over the non-productive DNA pol ε (Wu et al., 2021).

All three nuclear replicative DNA polymerases, α, δ, and ε, are components of the eukaryotic replication fork. Of note, these polymerases are multi-protein complexes, each of them comprising four different subunits (POLA1-4, POLD1-4, and POLE1-4, respectively); in all cases, subunit 1 carries catalytic activity, while subunit 2 has a regulatory function. DNA pol α initiates DNA replication by acting as the primase, assembling the RNA-DNA primers required for the processive DNA pol ε and δ to perform the bulk DNA synthesis on the leading and lagging strand, respectively. The primase subunits of DNA pol α, PRIM1 and PRIM2, are responsible for the *de novo* synthesis of a 7-12 ribonucleotide oligomer, which is subsequently extended with deoxyribonucleotides by DNA pol α (Pellegrini, 2012; Pedroza-Garcia et al., 2019).

In order to generate the dsDNA replicative intermediate, a CS needs to be synthesized in the first place. At least in some instances, the synthesis of the CS has been proposed to be initiated through the extension of a short RNA or DNA primer (Donson et al., 1984; Saunders et al., 1992); however, how general this strategy is remains to be determined. Given the recently demonstrated role of DNA pol α in the CS synthesis, and considering that this polymerase carries primase activity, we wondered whether the initiation of geminiviral replication required the action of PRIM1 and PRIM2, in addition to the requirement of POLA1 and POLA2 previously demonstrated (Wu et al., 2021).

With the aim of answering this question, we decided to silence *PRIM1* and *PRIM2* in *Nicotiana benthamiana* , and test the effect of this depletion on the replication of the geminivirus TYLCV. For this purpose, we first identified the PRIM1- and PRIM2-coding genes by homology of the resulting proteins with the Arabidopsis PRIM1 and PRIM2. As shown in Fig 1B and 1C, two orthologues were found in each case, which we named NbPRIM1-1/NbPRIM1-2 and NbPRIM2-1/NbPRIM2-2. We then generated constructs to silence each homologue pair by virus-induced gene silencing (VIGS) mediated by tobacco rattle virus (TRV); silencing was induced by infection with the TRV-based constructs, and silencing efficiency was measured 14 days later. A reduction in the abundance of target transcripts can be observed both in basal conditions and in the presence of TYLCV (as shown in Fig 1E). Knocking-down these genes, as expected, resulted in a visible developmental phenotype, which resembled that previously observed when silencing *NbPOLA1* or *NbPOLA2* (Supplementary Fig 4b; Wu et al., 2021), likely reflecting the requirement of DNA pol α for the replication of the plant genome.

Next, the effect of silencing *NbPRIM1-1/2* or *NbPRIM2-1/2* on the replication of TYLCV was tested in local infection assays in *N. benthamiana* leaves. In local *Agrobacterium* *tumefaciens* -mediated TYLCV infections, the accumulation of the viral DNA directly depends on the efficiency of viral DNA replication, and therefore can be used as a proxy for this process. Strikingly, upon silencing of either *NbPRIM1* or *NbPRIM2* , the accumulation of TYLCV was virtually abolished, as previously observed upon silencing of *NbPOLA1* or *NbPOLA2* (Wu et al., 2021) (Fig 1F), indicating that expression of these genes is required for the replication of the viral DNA.

These results do not necessarily indicate, however, that primase activity is required for geminiviral replication. It is conceivable that the DNA pol α holocomplex may get destabilized by the depletion of one of its subunits; in this scenario, the results presented here would only be an additional confirmation of the requirement of DNA pol α for the replication of the geminiviral DNA, but would shed no light on the specific requirement of the primase activity of the PRIM1/2 subunits. Nevertheless, a three-dimensional analysis of the *Saccharomyces cerevisiae* DNA pol α by electron microscopy indicates that the molecular architecture of this holocomplex consists of two physically independent but flexibly connected catalytic modules, that of the primase and that of the DNA polymerase, which enables primer transfer from the former to the latter (Núñez-Ramírez et al., 2011); interestingly, primer transfer can take place in mixtures of independent purified primase and polymerase complexes, demonstrating that both protein complexes can exist and retain activity in the absence of the other. Along these lines, human primase subunits expressed in *Escherichia coli* display primase activity, again supporting the idea that they can act independently of the other subunits of the DNA pol α complex (Schneider et al., 1998). It is therefore possible that POLA1 and POLA2 can still form a DNA replication-competent complex even in the absence of the primase subunits.

The results presented here demonstrate that PRIM1 and PRIM2, the primase subunits of DNA pol α, are required for the replication of TYLCV, as previously demonstrated for the catalytic and regulatory subunits of this holoenzyme; whether primase activity is required for the initiation of the viral CS during the viral replicative cycle remains however an open question to be investigated.

## Methods

**Plasmids and cloning**

The tomato yellow leaf curl virus-Almeria (TYLCV-Alm, Accession No. AJ489258) strain was used as template to generate the TYLCV infectious clone to destination vector pGWB501 (Rosas-Diaz et al., 2018). For virus-induced gene silencing (VIGS) assays, homologous genes of *PRIM1* and *PRIM2* were identified in *N. benthamiana* ( *NbPRIM1* -1/-2: Niben101Scf00366g01013, Niben101Scf01950g03015; *NbPRIM2* -1/-2: Niben101Scf13695g01002, Niben101Scf18347g00003). 300-bp conserved gene fragments were selected, PCR-amplified, and cloned into the pTRV2 vector (Liu et al., 2002) by traditional cloning using *Eco* RI and *Bam* HI High-Fidelity (HF®) restriction endonucleases (NEB) and T4 DNA ligase (NEB). In all cases, PCR products were amplified using Q5® High-Fidelity DNA Polymerase (NEB).

**Plant materials**

*N. benthamiana* plants used in this study are wild type, and were grown under long-day conditions (LD, 16 h of light/8 h of dark) at 25 °C.

**Virus-induced gene silencing (VIGS)**

Tobacco rattle virus (TRV)-mediated VIGS assays were performed as described in Medina-Puche et al., 2020. TRV-NbPDS targeting *Phytoene Desaturase* was used as a positive control. Briefly, *Agrobacterium* cells carrying pTRV1- and pTRV2-based constructs were grown in LB medium overnight with appropriate antibiotics. Cultures were resuspended in the infiltration buffer (10 mM MgCl _2_ , 10 mM MES, pH 5.6, and 150 µM acetosyringone) to an O.D. _600_ =0.5 and incubated at room temperature for 4 h in the dark. Mixed cell cultures were used to inoculate 2-week-old *N. benthamiana* plants on true leaves. Two weeks later, plants were used for local infection assays.

**Quantitative PCR (qPCR) and Reverse Transcription PCR (RT-qPCR)**

To determine viral accumulation, total DNA was extracted from infiltrated *N. benthamiana* leaves using the CTAB method (Minas et al., 2011). Quantitative PCR (qPCR) was performed with primers to amplify *Rep* . The 25S ribosomal DNA interspacer ( *ITS* ) was used as reference gene.

To detect gene expressions in *N. benthamiana* , total RNA was extracted from leaves by using Plant RNA kit (OMEGA Bio-tek). cDNA was synthesized using the iScript ^TM^ gDNA clear cDNA Synthesis Kit (Bio-Rad) according to the manufacturer’s instructions. *NbActin* was used as reference gene. qPCR and RT-qPCR were performed in a BioRad CFX96 real-time system with Hieff ^TM^ qPCR SYBR Green Master Mix (Yeason). The reactions were done as follows: 3 min at 95 °C, 40 cycles consisting of 15 s at 95 °C, 30 s at 60 °C.

**Local TYLCV infection**

Local TYLCV viral infection assays were done as described in Wu *et al* ., 2019. In brief, the *Agrobacterium* clones harbouring the infectious clone of TYLCV or an empty vector (EV) as control were liquid-cultured in LB with appropriate antibiotics overnight. Bacterial cultures were centrifuged at 4000 × g for 10 min and resuspended in infiltration buffer (10 mM MgCl _2_ , 10 mM MES, pH 5.6, and 150 µM acetosyringone) to an O.D. _600_ =0.5. After a 4-h incubation at room temperature in the dark, bacterial cultures were used to infiltrate the underside of leaves of 4-week-old *N. benthamiana* plants.

## Reagents

**Table 1: Primers used in this study.**

**Table:** 

		
**Primers**	**5’ ~ 3’ Sequence**	**Purpose**
** TRV2: *NbPRIM1* -1/2 **	F: CGGAATTCCGAAGCTAAGAGAGGATCATC R: CGGGATCCCGTCTCAAATTCTCCTCAC	Cloning
** TRV2: *NbPRIM2* -1/2 **	F: CGGAATTCCGCAGGAGCTGATGTTTGTTTG R: CGGGATCCCGCTTGTAAGACATCAGTA	
***25S ribosomal DNA interspacer (ITS)***	F: ATAACCGCATCAGGTCTCCA R: CCGAAGTTACGGATCCATTT	RT-qPCR
***NbACTIN***	F: CGGAATCCACGAGACTACATAC R: GGGAAGCCAAGATAGAGC	
***Rep (TYLCV)***	F: TGAGAACGTCGTGTCTTCCG R: TGACGTTGTACCACGCATCA	
***NbPRIM1* -1/2 **	F: GCTCCAAACCCCTTAAGCCA R: GAAACCACCTGGCTCATTGC	
***NbPRIM2* -1/2 **	F: TCATACTCTGGTCGGAGGGA R: CGGAAATATCCTCCAAGCTGT	
